# L-shaped relationship between stress hyperglycemia ratio and cardiovascular disease risk in middle-aged and older adults: Insight from the China Health and Retirement Longitudinal Study

**DOI:** 10.1371/journal.pone.0324978

**Published:** 2025-05-20

**Authors:** Zixi Zhang, Jiabao Zhou, Cancan Wang, Kang Wang, Yongguo Dai, Qiuzhen Lin, Yichao Xiao, Qiming Liu

**Affiliations:** 1 Department of Cardiology, The Second Xiangya Hospital, Central South University, Changsha, Hunan, People’s Republic of China; 2 Department of Endocrinology, The Second Xiangya Hospital, Central South University, Changsha, Hunan, People’s Republic of China; 3 Department of Pharmacy, Xiangya Hospital, Central South University, Changsha, Hunan, People’s Republic of China; Albert Einstein College of Medicine, UNITED STATES OF AMERICA

## Abstract

**Background:**

The stress hyperglycemia ratio (SHR) has emerged as a potential marker for predicting cardiovascular disease (CVD) and mortality. However, its relationship with CVD and all-cause mortality in middle-aged and older Chinese populations remains unclear.

**Methods:**

This study analyzed data from the China Health and Retirement Longitudinal Study (CHARLS), including individuals aged 45 years and older. Cross-sectional analysis assessed the associations between SHR and CVD incidence, whereas longitudinal Cox regression models evaluated the relationships between SHR, CVD risk, and all-cause mortality. Restricted cubic spline analyses were employed to explore potential non-linear relationships. The predictive performance of SHR was compared with that of fasting blood glucose (FBG) and glycosylated hemoglobin (HbA1c).

**Results:**

The cross-sectional analysis identified an inverse association between the SHR and CVD incidence. Longitudinal analysis indicated that SHR was independently associated with an increased risk of CVD, with a significant L-shaped relationship (non-linear *P* = 0.001). Threshold effect analysis identified 0.985 as the inflection point for SHR, with hazard ratios (HRs) increasing sharply below this level (HR: 0.32, 95% CI: 0.17–0.61, *P* = 0.001). However, no significant non-linear relationship was observed between SHR and all-cause mortality (non-linear *P* = 0.942). FBG, HbA1c, and SHR provided similar predictive value for all-cause mortality (area under the curve: 0.526 vs. 0.535 vs. 0.513), without significant incremental predictive value.

**Conclusions:**

The SHR is an independent predictor of CVD risk in middle-aged and older Chinese adults, with an L-shaped relationship. Future large-scale, multicenter studies are needed to validate these findings.

## 1 Introduction

Cardiovascular diseases (CVDs) remain the leading cause of mortality worldwide, accounting for approximately 32% of all deaths [1]. The burden of CVD is increasing most rapidly in low- and middle-income countries, driven by population aging, urbanization, and lifestyle transitions. In China, CVDs are the primary cause of death, responsible for more than 40% of all disease-related mortality [2]. These trends underscore the urgent need for more refined risk stratification tools and targeted prevention strategies to mitigate the growing public health impact.

Dysglycemia—encompassing both chronic hyperglycemia and acute glucose fluctuations—has been recognized as a critical pathophysiological mediator in CVD development. Chronic hyperglycemia promotes endothelial dysfunction through the accumulation of advanced glycation end-products, while acute stress-induced hyperglycemia (SIH) increases plaque vulnerability via matrix metalloproteinase-9 activation, thereby accelerating atherogenesis [3,4]. However, conventional glycemic indices such as fasting blood glucose (FBG) and glycated hemoglobin (HbA1c) are insufficient to capture these dynamic fluctuations. For instance, the data from China Kadoorie Biobank revealed that 41% of incident strokes occurred in individuals with HbA1c levels < 5.7%, indicating substantial residual risk undetected by standard markers of glycemic status [5]. To address this limitation, the stress hyperglycemia ratio (SHR), calculated as $\frac{FBG}{\left(1.59\times HbA1c-2.59\right)}$, has been proposed as a composite marker that integrates acute and chronic glycemic states [6]. Elevated SHR has been associated with adverse outcomes in patients with acute myocardial infarction (AMI), chronic total occlusion, and in individuals with prediabetes or type 2 diabetes mellitus [7,8]. These findings support the potential clinical utility of SHR as a more sensitive and integrative marker for cardiovascular risk assessment compared to traditional glycemic indicators.

Despite the growing evidence, most SHR-related studies have been conducted in Western populations [9–11]. The applicability of SHR in East Asian populations remains underexplored. This knowledge gap was particularly important given the distinct cardiometabolic profiles in East Asians, including heightened β-cell susceptibility, high-glycemic-index dietary patterns (e.g., rice-based diets), and exposure to environmental stressors such as air pollution—all of which may influence postprandial glucose dynamics and CVD risk [12–15]. Furthermore, while SHR has demonstrated utility as a predictor of CVD and mortality in diabetic populations, its role in non-diabetic individuals remains unclear.

To address these gaps, we conducted a population-based study using data from the China Health and Retirement Longitudinal Study (CHARLS), a nationally representative cohort of middle-aged and older adults. We assessed the cross-sectional and longitudinal associations between SHR, prevalent CVD, and all-cause mortality. In addition, we explored potential non-linear relationships between SHR and cardiovascular outcomes. These findings were intended to clarify the role of SHR in cardiovascular risk stratification and inform targeted prevention strategies for high-risk individuals in East Asian populations.

## 2 Methods

### 2.1 Study design and population

The data utilized in this study were extracted from the CHARLS, a nationally representative, ongoing longitudinal survey established in 2011. The CHARLS collects high-quality data through face‒to‒face interviews using structured questionnaires from Chinese individuals aged 45 years and older. A multistage stratified probability‒proportional‒to‒size sampling method was employed, and participants were assessed via standardized questionnaires to gather data on sociodemographic characteristics, lifestyle factors, and health-related information. Following the baseline survey, all participants underwent followed up assessments every 2–3 years, and new participants were recruited. Detailed information regarding the study design of the CHARLS has been previously reported [16].

**Fig 1:**
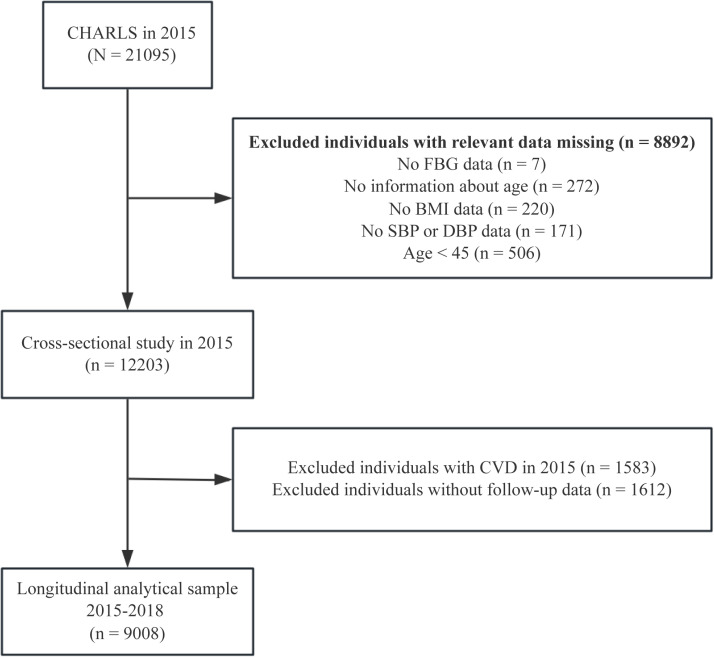
Flow diagram for participants included in the study. Abbreviations: BMI, body mass index; CVD, cardiovascular disease; DBP, diastolic blood pressure; FBG, fasting blood glucose; SBP, systolic blood pressure.

We downloaded the data from the CHARLS website (https://charls.pku.edu.cn/) for the 2015 and 2018 waves. The inclusion criterion for this study was as follows: (i) all individuals included in CHARLS 2015. The exclusion criteria were as follows: (i) missing FBG data; (ii) missing HbA1c data; (iii) missing age information; (iv) absence of body mass index (BMI) data; (v) absence of blood pressure data; and (vi) age < 45 years. This study was divided into two sections: (1) In the cross-sectional analysis, we used data from the large cohort that was followed up in CHARLS 2015. A total of 21095 participants were interviewed in CHARLS 2015; 8892 individuals were excluded because of missing data on FBG (n = 7716) and HbA1c (n = 7), no information about age (n = 272), lack of BMI (n = 220) and blood pressure data (n = 171), and aged less than 45 years (n = 506), leaving 12203 participants for cross-sectional analysis. (2) In the longitudinal analysis, we further excluded 1583 subjects with CVD from the CHARLS 2015 and 1612 participants without follow-up data from the CHARLS 2018. Our ﬁnal analytical sample consisted of 9008 individuals who had no CVD in CHARLS 2015 and were completely followed up in 2018. The detailed participant selection process is shown in Fig 1.

All participants provided informed consent, and the study protocol was approved by the ethical review committee of Peking University (approval number: IRB00001052 − 11015). All procedures performed in studies involving human participants were in accordance with the ethical standards of the institutional and/or national research committee and with the 1964 Helsinki declaration and its later amendments or comparable ethical standards. This study was conducted following the Strengthening the Reporting of Observational Studies in Epidemiology (STROBE) reporting guideline [17].

### 2.2 Assessment of SHR

The SHR was calculated using the formula: $\begin{array}{c} SHR=\frac{[FBG\;(\frac{mmol}{L})]}{[1.59\times HbA1c\;(\%)-2.59]} \end{array}$ [18]. In the CHARLS, FBG was measured using an enzymatic colorimetric test, and HbA1c was measured using boronated affinity liquid chromatography on the basis of overnight fasting of venous blood samples (https://charls.charlsdata.com/pages/data/111/en.html). The protocol of the blood-based biomarker sample collection study was approved by the ethical review committee of Peking University (IRB00001052 − 11014). Written informed consent was obtained from all participants. All patients were classified into four groups (Q1, Q2, Q3, and Q4) by quartile of SHR, with Q1 as the reference group.

### 2.3 Assessment of CVD incidence

The study outcome was the incidence of CVD, including heart disease and stroke. Consistent with previous studies [19], these conditions were assessed through the following questions: “Have you been diagnosed with heart attack, coronary heart disease, angina, congestive heart failure, or other heart problems by a doctor?” (Code: zda007_7_), and “Have you been diagnosed with stroke by a doctor?” (zda007_8_:). Participants who reported either heart disease or stroke were deﬁned as having CVD.

### 2.4 Covariates

At baseline, trained interviewers collected information on sociodemographic status and health-related factors using a structured questionnaire. Sociodemographic variables included age, sex, and marital status (categorized as married or other). Health-related factors included BMI, mean systolic blood pressure (SBP), mean diastolic blood pressure (DBP), alcohol consumption (categorized as drinker or non-drinker), hypertension (zda007_1_), diabetes (zda007_3_), self-reported physician-diagnosed dyslipidemia (zda007_2_) and kidney disease (zda007_9_), and the use of medications for hypertension (da011s1; da011s2; da011s3), diabetes (da014s1; da014s2; da014s3), and dyslipidemia (da010_2_s1; da010_2_s2; da010_2_s3). Blood pressure was averaged from three consecutive measurements. Hypertension was defined as a mean SBP ≥ 140 mmHg, mean DBP ≥ 90 mmHg, or current use of antihypertensive medication. Diabetes was identified on the basis of the use of insulin or oral hypoglycemic agents or a plasma glucose level ≥ 200 mg/dL. In the cross-sectional analysis, metabolic biomarkers, including total cholesterol (TC), triglyceride (TG), serum creatinine, uric acid (UA), and C-reactive protein (CRP), were measured in 12203 individuals. The methods used to measure TC, TG, serum creatinine, UA, and CRP, have been previously described in detail (https://charls.pku.edu.cn/en/). The estimated glomerular filtration rate (eGFR) was calculated using the Chronic Kidney Disease Epidemiology Collaboration’s 2009 creatinine equation [20].

### 2.5 Statistical analysis

Baseline characteristics were stratified by the SHR quartiles. Continuous variables were presented as the means ± standard deviations, whereas categorical variables are expressed as frequency counts and percentages. The *P* values for continuous variables were calculated via one-way ANOVA, and for categorical variables, Pearson’s chi-square test was employed. In the cross-sectional analysis, logistic regression was used to estimate the association between SHR and CVD incidence. For the longitudinal analysis, we calculated the incidence of CVD and all-cause mortality using the 2018 CHARLS data. Since the CHARLS database does not provide detailed follow-up data for each individual, the follow-up period was defined as the time from the baseline interview to either the diagnosis of CVD, the last follow-up interview (March 2019), or death. Multivariable Cox proportional hazards models were utilized to assess the prognostic value of the SHR. Three Cox models were constructed with different levels of adjustment: Model 1 adjusted for age and sex; Model 2 further adjusted for marital status, BMI, and alcohol consumption; and Model 3 included additional adjustments for mean SBP and DBP, hypertension, dyslipidemia, diabetes, chronic kidney disease, medication use (antihypertensive, antidiabetic, and lipid-lowering), TC, TG, eGFR, UA, and CRP. These covariates were selected based on their known associations with CVD and their significant differences across SHR quartiles, as observed in the baseline analysis.

To explore the associations between SHR, CVD, and all-cause mortality, we employed restricted cubic spline (RCS) analysis within a Cox proportional hazards model framework, adjusting for the same confounders as in the Cox models. The number of knots was set to 3. If a non-linear association was detected, the threshold value was estimated, and the inflection point with the highest likelihood was identified. A two-piecewise Cox proportional hazards model was then applied on both sides of the inflection point to investigate the association between the SHR and CVD incidence. Subgroup analyses were conducted to assess the impact of SHR on CVD and all-cause mortality, stratified by age (< 60 years vs. ≥ 60 years), sex, alcohol consumption (heavy, mild, never), BMI (< 24 kg/m^2^ vs. ≥ 24 kg/m^2^), diabetes, and kidney disease. Receiver operating characteristic (ROC) curve analysis was performed to compare the predictive value of FBG, HbA1c, and SHR in assessing the risk of all-cause mortality. The area under the curve (AUC) was evaluated using the DeLong test. Subsequently, each of the three markers was incorporated into a baseline risk model (Cox regression Model 3) to assess their incremental predictive value. All the statistical analyses were performed via R 4.3.2 (R Foundation for Statistical Computing). A *P* value < 0.05 was considered statistically significant.

## 3 Results

### 3.1 Baseline characteristics of participants in the cross‑sectional and longitudinal analyses

A total of 12203 individuals were included in the cross-sectional study (Table 1). The mean age was 60.6 ± 9.7 years, with 47.2% of the participants were male. The mean SHR values for quartiles Q1, Q2, Q3, and Q4 were 0.666, 0.771, 0.844, and 1.047, respectively. Participants with higher SHR values tended to be older, female, non-drinkers, and have a lower BMI. Hypertension was the most prevalent comorbidity (22.0%), while the incidence of heart disease and stroke were 11.4% and 2.1%, respectively. Significant interquartile differences were observed in metabolic biomarkers (*P* < 0.01), with higher TG and UA levels in the highest SHR quartile. Detailed baseline characteristics of 9008 participants without CVD included in the longitudinal cohort are summarized in S1 Table.

**Table 1:** Baseline characteristics of the four groups.

Characteristics	Q1 (≤ 0.737)	Q2 (0.737–0.805]	Q3 (0.805–0.888]	Q4 (> 0.888)	Total	*P* value
	n = 3036	n = 3076	n = 3063	n = 3028	N = 12203	
SHR	0.666 ± 0.066	0.771 ± 0.019	0.844 ± 0.024	1.047 ± 0.199	0.832 ± 0.174	
**Demographics**						
Age (y)	61.1 ± 9.6	61.0 ± 9.5	60.0 ± 9.6	60.2 ± 9.9	60.6 ± 9.7	< 0.001
Male (n, %)	1402 (46.2)	1382 (44.9)	1404 (45.8)	1574 (52.0)	5762 (47.2)	< 0.001
Marry (n, %)	2647 (87.2)	2669 (86.8)	2660 (86.8)	2622 (86.6)	10598 (86.8)	0.920
**Basic information**						
Alcohol consumption	995 (32.8)	988 (32.2)	1135 (37.1)	1191 (39.4)	4309 (35.4)	< 0.001
SBP (mmHg)	127.4 ± 20.6	128.5 ± 19.9	128.1 ± 19.2	130.0 ± 19.7	128.5 ± 19.9	< 0.001
DBP (mmHg)	75.1 ± 11.8	75.7 ± 11.7	75.8 ± 11.3	76.4 ± 11.9	75.7 ± 11.7	<0.001
Height (cm)	158.7 ± 8.4	158.0 ± 8.5	158.3 ± 8.6	158.9 ± 8.6	158.2 ± 8.6	< 0.001
Weight (kg)	59.0 ± 12.1	59.7 ± 11.6	60.4 ± 11.7	61.6 ± 12.2	60.2 ± 11.9	< 0.001
BMI (kg/m^2^)	23.7 ± 4.3	23.9 ± 3.8	24.1 ± 4.0	24.3 ± 4.1	24.0 ± 4.1	<0.001
**Comorbidities**						
Diabetes (n, %)	186 (6.1)	125 (4.1)	145 (4.7)	227 (7.5)	683 (5.6)	<0.001
Hypertension (n, %)	650 (21.4)	693 (22.5)	633 (20.7)	709 (23.4)	2685 (22.0)	0.050
Dyslipidemia (n, %)	289 (9.5)	333 (10.8)	271 (8.8)	303 (10.0)	1196 (9.8)	0.065
Heart disease (n, %)	406 (13.4)	357 (11.6)	301 (9.8)	328 (10.8)	1392 (11.4)	< 0.001
Stroke (n, %)	78 (2.6)	56 (1.8)	56 (1.8)	67 (2.2)	257 (2.1)	0.128
Kidney disease (n, %)	194 (6.4)	191 (6.2)	219 (7.1)	161 (5.3)	765 (6.3)	0.032
**Medical treatment**						
Diabetes medications (n, %)	218 (7.2)	118 (3.8)	151 (4.9)	289 (9.5)	776 (6.4)	< 0.001
Hypertension medications (n, %)	928 (30.6)	978 (31.8)	963 (31.4)	1051 (34.7)	3920 (32.1)	0.004
Lipid-lowering therapy	248 (8.2)	253 (8.2)	254 (8.3)	264 (8.7)	1019 (8.4)	0.863
**Laboratory data**						
TC (mg/dL)	183.8 ± 37.0	185.8 ± 35.1	185.1 ± 35.5	181.2 ± 36.6	184.0 ± 36.1	< 0.001
TG (mg/dL)	126.5 ± 78.0	134.3 ± 81.0	144.0 ± 91.4	168.0 ± 106.2	143.2 ± 91.1	< 0.001
eGFR (mL/min/1.73m^2^)	89.7 ± 15.7	89.9 ± 15.9	90.6 ± 15.9	90.0 ± 17.6	90.0 ± 16.3	0.008
UA (umol/L)	4.9 ± 1.4	4.9 ± 1.4	5.0 ± 1.4	5.1 ± 1.5	5.0 ± 1.4	< 0.001
CRP (mg/L)	3.0 ± 7.2	2.4 ± 5.0	2.7 ± 6.0	2.8 ± 5.1	2.7 ± 5.9	< 0.001

The values are presented as the means ± standard deviations or n (%). A *P* value < 0.05 indicated a significant difference.

Abbreviations: BMI, body mass index; CRP, C-reactive protein; CVD, cardiovascular disease; DBP, diastolic blood pressure; eGFR, estimated glomerular filtration rate; FBG, fasting blood glucose; SBP, systolic blood pressure; SHR, stress hyperglycemia ratio; TC, total cholesterol; TG, triglyceride; UA, uric acid.

### 3.2 Cross-sectional associations between SHR and CVD

In the cross-sectional analysis, the prevalence of CVD decreased across SHR quartiles (Q1–Q4: 15.4%, 13.0%, 11.3%, and 12.2%; *P* < 0.001). After multivariable adjustment, participants in Q2 [odds ratio (OR): 0.78, 95% confidence interval (CI): 0.66 − 0.91], Q3 (OR:0.69, 95% CI: 0.59 − 0.81), and Q4 (OR:0.71, 95% CI: 0.61 − 0.84) were significantly associated with CVD (all *P* < 0.001) (Table 2).

**Table 2:** Cross-sectional association between the quartile of SHR, CVD and its components among all participants.

Outcome	cases, n (%)	OR (95% CI) *P* value		
		Model 1	Model 2	Model 3
**CVD**				
Q1 (n = 3036)	468 (15.4)	Reference	Reference	Reference
Q2 (n = 3076)	400 (13.0)	0.82 (0.71 − 0.94) 0.006	0.81 (0.70 − 0.94) 0.004	0.78 (0.66 − 0.91) 0.001
Q3 (n = 3063)	345 (11.3)	0.72 (0.62 − 0.84) < 0.001	0.71 (0.61 − 0.83) < 0.001	0.69 (0.59 − 0.81) < 0.001
Q4 (n = 3028)	370 (12.2)	0.80 (0.69 − 0.92) 0.003	0.76 (0.66 − 0.89) < 0.001	0.71 (0.61 − 0.84) < 0.001
Total (n = 12203)	1583 (13.0)	0.57 (0.41 − 0.79) < 0.001	0.51 (0.36 − 0.71) < 0.001	0.45 (0.32 − 0.64) < 0.001
**Heart diseases**				
Q1 (n = 3036)	406 (13.4)	Reference	Reference	Reference
Q2 (n = 3076)	357 (11.6)	0.85 (0.73 − 0.99) 0.034	0.84 (0.72 − 0.98) 0.027	0.81 (0.68 − 0.95) 0.009
Q3 (n = 3063)	301 (9.8)	0.73 (0.62 − 0.86) < 0.001	0.72 (0.61 − 0.85) < 0.001	0.70 (0.59 − 0.82) < 0.001
Q4 (n = 3028)	328 (10.8)	0.82 (0.70 − 0.96) 0.015	0.79 (0.67 − 0.92) 0.003	0.74 (0.62 − 0.87) < 0.001
Total (n = 12203)	1392 (11.4)	0.55 (0.39 − 0.79) 0.001	0.50 (0.35 − 0.71) < 0.001	0.44 (0.30 − 0.63) < 0.001
**Stroke**				
Q1 (n = 3036)	78 (2.6)	Reference	Reference	Reference
Q2 (n = 3076)	56 (1.8)	0.71 (0.50 − 1.00) 0.051	0.70 (0.49 − 1.00) 0.047	0.68 (0.47 − 0.98) 0.038
Q3 (n = 3063)	56 (1.8)	0.75 (0.53 − 1.06) 0.101	0.72 (0.51 − 1.03) 0.073	0.76 (0.53 − 1.09) 0.135
Q4(n = 3028)	67 (2.2)	0.88 (0.63 − 1.23) 0.456	0.86 (0.61 − 1.20) 0.376	0.81 (0.57 − 1.16) 0.254
Total (n = 12203)	257 (2.1)	1.35 (0.70 − 2.59) 0.371	1.25 (0.64 − 2.44) 0.508	1.16 (0.60 − 2.25) 0.655

A *P* value < 0.05 indicated a significant difference.

Abbreviation: BMI, body mass index; CI, confidence interval; CRP, C-reactive protein; CVD, cardiovascular disease; DBP, diastolic blood pressure; eGFR, estimated glomerular filtration rate; OR, odds ratio; SBP, systolic blood pressure; SHR, stress hyperglycemia ratio; TC, total cholesterol; TG, triglycerides; UA, uric acid.

Model 1: Adjusted for age and sex.

Model 2: Adjusted for age, sex, marital status, BMI, and alcohol consumption.

Model 3: Adjusted for age, sex, marital status, BMI, alcohol consumption, mean SBP and DBP, hypertension, dyslipidemia, diabetes, chronic kidney disease, use diabetes medications, use hypertension medications, lipid-lowering therapy, TC, TG, eGFR, UA, and CRP.

**Fig 2:**
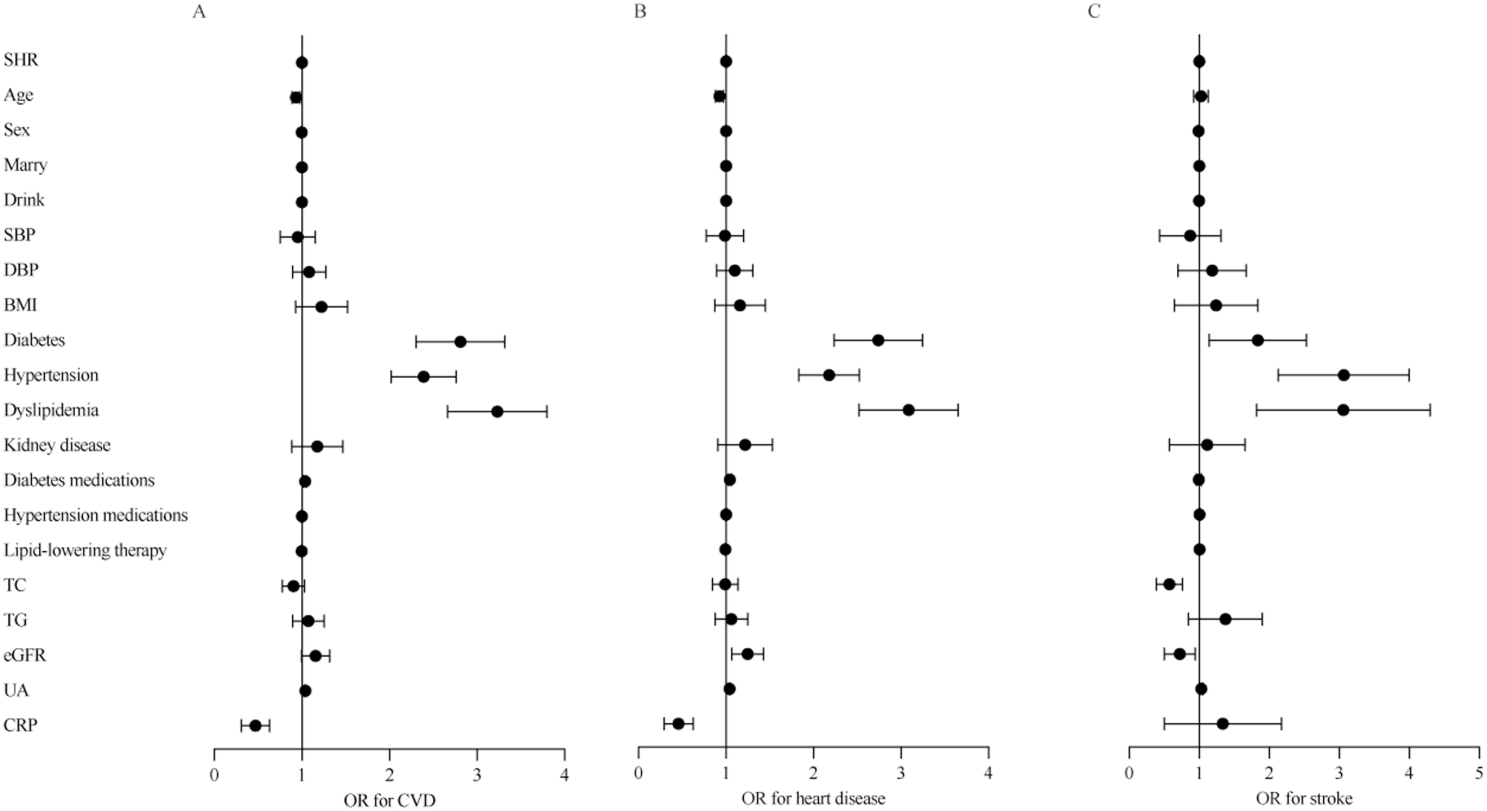
ORs and 95% CIs of CVD incidence and its components according to the SHR in the cross-sectional analysis. Forest plots show ORs and 95% CIs for A) CVD, B) heart disease, and C) stroke adjusted for age, sex, marital status, drinking status, BMI, SBP, and DBP; history of diabetes, hypertension, dyslipidemia, and chronic kidney disease; use diabetes medications; use hypertension medications; use of lipid-lowering therapy; TC; TG; eGFR; UA; and CRP. Abbreviations: BMI, body mass index; CI, confidence interval; CRP, C-reactive protein; CVD, cardiovascular disease; DBP, diastolic blood pressure; eGFR, estimated glomerular filtration rate; OR, odds ratio; SBP, systolic blood pressure; SHR, stress hyperglycemia ratio; TC, total cholesterol; TG, triglyceride; UA, uric acid.

Table 2 also illustrates the associations between the SHR and different CVD components. After adjusting for covariates, Q2 (OR: 0.81, 95% CI: 0.68 − 0.95, *P* = 0.009), Q3 (OR: 0.70, 95% CI: 0.59 − 0.82, *P* < 0.001), and Q4 (OR: 0.74, 95% CI: 0.63 − 0.87, *P* < 0.001) showed significant negative associations with heart disease. Although Q3 and Q4 were negatively associated with stroke, these associations were not statistically significant (*P* = 0.135; *P* = 0.254). In a subgroup of 12203 participants who completed metabolic biomarker measurements, similar results were observed after further adjustment for metabolic biomarkers. SHR was significantly associated with CVD (OR: 0.45, 95% CI: 0.32 − 0.64, *P* < 0.001) and heart disease (OR: 0.44, 95% CI: 0.30 − 0.63, *P* < 0.001), but not with stroke (*P* = 0.655) (Fig 2) (S2 Table).

### 3.3 Longitudinal association among SHR, CVD incidence and all-cause mortality

During a mean follow-up period of 42.2 ± 1.1 months, 1085 incident CVD events and 229 deaths from all causes were recorded, yielding incidence rates of 120.4 and 25.4 per 1000 person-years, respectively. Table 3 shows the three Cox regression models used to evaluate the correlation between the SHR and CVD events. In Model 1, the hazard ratios (HRs) and CIs for quartiles Q1 to Q4 were 1.00 (reference), 0.90 (0.77 − 1.06), 0.81 (0.68 − 0.95), and 0.86 (0.73 − 1.01), with a *P* value < 0.05 for Q3. In Model 3, the HRs were 1.00 (reference), 0.89 (0.76–1.05), 0.79 (0.66–0.93), and 0.79 (0.67–0.94), respectively, with *P* values < 0.05 for Q3 and Q4.

**Table 3:** Cox regression models for the association between the SHR, CVD incidence, and all-cause mortality.

	Continuous variable	Quantiles of the SHR				*P* for trend
		Q1	Q2	Q3	Q4	
**CVD events**						
Model 1 HR (95% CI) *P* value	0.69 (0.47–1.01) 0.057	1(Ref)	0.90 (0.77–1.06) 0.220	0.81 (0.68–0.95) 0.012	0.86 (0.73–1.01) 0.069	0.029
Model 2 HR (95% CI) *P* value	0.64 (0.43–0.93) 0.021	1(Ref)	0.89 (0.76–1.05) 0.167	0.79 (0.67–0.94) 0.007	0.83 (0.70–0.98) 0.024	0.010
Model 3 HR (95% CI) *P* value	0.56 (0.38–0.82) 0.003	1(Ref)	0.89 (0.76–1.05) 0.168	0.79 (0.66–0.93) 0.005	0.79 (0.67–0.94) 0.006	0.002
**All-cause mortality**						
Model 1 HR (95% CI) *P* value	0.83 (0.43–1.60) 0.583	1(Ref)	1.07 (0.74–1.53) 0.723	0.93 (0.63–1.36) 0.705	0.95 (0.68–1.34) 0.779	0.656
Model 2 HR (95% CI) *P* value	0.83 (0.42–1.62) 0.578	1(Ref)	1.06 (0.74–1.53) 0.744	0.93 (0.63–1.37) 0.721	0.93 (0.65–1.33) 0.700	0.589
Model 3 HR (95% CI) *P* value	0.88 (0.43–1.78) 0.718	1(Ref)	1.10 (0.75–1.62) 0.620	0.95 (0.63–1.44) 0.815	0.97 (0.67–1.40) 0.867	0.764

A *P* value < 0.05 indicated a significant difference.

Abbreviations: BMI, body mass index; CI, confidence interval; CRP, C-reactive protein; CVD, cardiovascular disease; DBP, diastolic blood pressure; eGFR, estimated glomerular filtration rate; HR, hazard ratio; SBP, systolic blood pressure; SHR, stress hyperglycemia ratio; TC, total cholesterol; TG, triglyceride; UA, uric acid.

Model 1: Adjusted for age and sex.

Model 2: Adjusted for age, sex, marital status, BMI, and alcohol consumption.

Model 3: Adjusted for age, sex, marital status, BMI, alcohol consumption, mean SBP and DBP, hypertension, dyslipidemia, diabetes, chronic kidney disease, use diabetes medications, use hypertension medications, lipid-lowering therapy, TC, TG, eGFR, UA, and CRP.

Table 3 also shows the Cox regression models for all-cause mortality. After adjusting only for age and sex, the HRs and 95% CIs for Q1 to Q4 were 1.00 (reference), 1.07 (0.74–1.53), 0.93 (0.63–1.36), and 0.95 (0.68–1.34), respectively. In Model 3, the HRs were 1.00 (reference), 1.10 (0.75–1.62), 0.95 (0.63–1.44), and 0.97 (0.67–1.40), respectively, with no statistically significant *P* values across Q2 to Q4.

### 3.4 Non-linear relationships between the SHR, CVD incidence, and all-cause mortality

**Fig 3:**
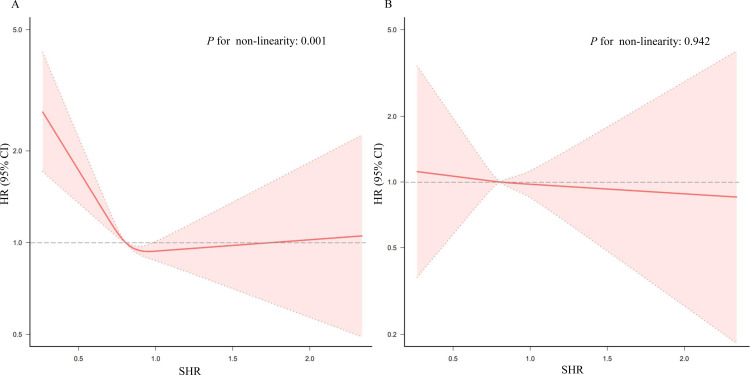
Association between the SHR and CVD (A) and all-cause mortality (B). Adjusted for age, sex, marital status, drinking status, BMI, SBP, and DBP; history of diabetes, hypertension, dyslipidemia, and chronic kidney disease; use diabetes medications; use hypertension medications; use of lipid-lowering therapy; TC; TG; eGFR; UA; and CRP. The solid line and pink area represent the estimated values and their corresponding 95% CIs, respectively. A *P* value < 0.05 was considered statistically significant. Abbreviations: BMI, body mass index; CI, confidence interval; CRP, C-reactive protein; CVD, cardiovascular disease; DBP, diastolic blood pressure; eGFR, estimated glomerular filtration rate; HR, hazard ratio; SBP, systolic blood pressure; SHR, stress hyperglycemia ratio; TC, total cholesterol; TG, triglyceride; UA, uric acid.

The RCS analysis revealed an L-shaped relationship between SHR and CVD after adjusting for covariates (non-linearity *P* value  < 0.05) (Fig 3A). The risk of CVD increased sharply when SHR was below 0.985 and plateaued thereafter (Fig 3A). A two-piecewise Cox model confirmed the inflection point at SHR = 0.985. When the SHR was < 0.985, the risk of CVD increased as the SHR decreased (HR: 0.32, 95% CI: 0.17–0.61, *P* = 0.001) (Table 4). However, no significant non-linear relationship was found between SHR and all-cause mortality (*P* = 0.942) (Fig 3B).

**Table 4:** Threshold effect analysis of the SHR on CVD events.

	Adjusted HR (95% CI)	*P* value
**CVD events**		
Fitting by two-piecewise Cox proportional risk model		
Inflection point	0.985	
SHR index < 0.985	0.32 (0.17–0.61)	0.001
SHR index ≥ 0.985	1.53 (0.63–3.71)	0.349
*P* for Log-likelihood ratio		0.013

The model was adjusted for age, sex, marital status, BMI, alcohol consumption, mean SBP and DBP, hypertension, dyslipidemia, diabetes, chronic kidney disease, use diabetes medications, use hypertension medications, lipid-lowering therapy, TC, TG, eGFR, UA, and CRP. A *P* value < 0.05 indicated a significant difference.

Abbreviations: BMI, body mass index; CI, confidence interval; CRP, C-reactive protein; CVD, cardiovascular disease; DBP, diastolic blood pressure; eGFR, estimated glomerular filtration rate; HR, hazard ratio; SBP, systolic blood pressure; SHR, stress hyperglycemia ratio; TC, total cholesterol; TG, triglyceride; UA, uric acid.

### 3.5 Subgroup analysis

Subgroup analyses showed consistent associations between SHR and CVD incidence across all strata, including age, sex, BMI, alcohol consumption, diabetes, and kidney disease. No significant interactions were observed between SHR and any subgroup variables (Table 5).

**Table 5:** Subgroup analyses of the association between the SHR and CVD incidence.

SHR	HR (95% CI)		*P* value	*P* interaction
	<0.985	≥ 0.985		
**Overall**				
**Age, years**				0.694
<60	Reference	0.78 (0.56–1.07)	0.125	
≥60	Reference	0.82 (0.64–1.05)	0.109	
**Sex**				0.384
Female	Reference	0.74 (0.55–0.98)	0.034	
Male	Reference	0.88 (0.67–1.15)	0.353	
**Drink**				0.264
Heavy	Reference	0.82 (0.64–1.04)	0.108	
Mild	Reference	0.69 (0.46–1.03)	0.072	
Never	Reference	1.23 (0.69–2.17)	0.480	
**BMI**				0.158
< 24	Reference	0.91 (0.68–1.20)	0.499	
≥ 24	Reference	0.72 (0.55–0.95)	0.019	
**Diabetes**				0.318
Yes	Reference	1.05 (0.59–1.86)	0.865	
No	Reference	0.78 (0.63–0.96)	0.018	
**Kidney disease**				0.108
Yes	Reference	1.34 (0.65–2.75)	0.429	
No	Reference	0.78 (0.64–0.95)	0.016	

A *P* value < 0.05 indicated a significant difference.

Abbreviations: BMI, body mass index; CI, confidence interval; CVD, cardiovascular disease; HR, hazard ratio; SHR, stress hyperglycemia ratio.

### 3.6 ROC analysis

**Fig 4:**
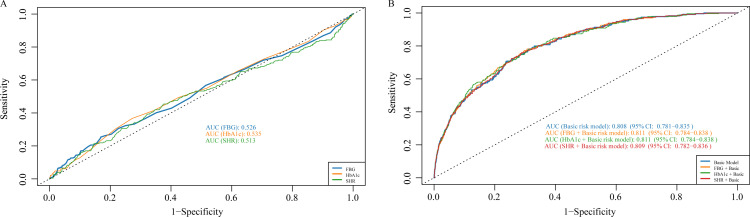
ROC curve analysis of metabolic markers for predicting all-cause mortality. Adjusted for age, sex, marital status, BMI, alcohol consumption, mean SBP and DBP, hypertension, dyslipidemia, diabetes, chronic kidney disease, use diabetes medications, use hypertension medications, lipid-lowering therapy, TC, TG, eGFR, UA, and CRP. A *P* value < 0.05 was considered statistically significant. Abbreviations: AUC, area under the curve; BMI, body mass index; CRP, C-reactive protein; DBP, diastolic blood pressure; eGFR, estimated glomerular filtration rate; ROC, receiver operating characteristic; SBP, systolic blood pressure; SHR, stress hyperglycemia ratio; TC, total cholesterol; TG, triglyceride; UA, uric acid.

ROC curves analyses indicated that no significant difference was observed in the AUCs between HbA1c and SHR compared to FBG (0.526 vs. 0.535 vs. 0.513) (Fig 4A, S3 Table). Furthermore, we assessed whether incorporating FBG, HbA1c, and SHR could enhance the predictive value of the baseline risk model. As shown in Fig 4B, none of these markers provided significant incremental predictive value.

## 4 Discussion

To the best of our knowledge, this is the first study to investigate the association between the SHR, CVD incidence, and all-cause mortality among middle-aged and elderly Chinese individuals. The major findings of our study were as follows: (1) SHR was inversely associated with CVD in both cross-sectional and longitudinal analyses, particularly with heart disease; (2) an L-shaped relationship was observed between SHR and CVD, with significantly increased risk when SHR < 0.985; (3) SHR was not significantly associated with all-cause mortality; and (4) SHR, FBG, and HbA1c demonstrated comparable predictive value for mortality, without significant incremental predictive value.

The SHR is a novel indicator of SIH and is recognized as an effective marker for assessing illness severity in critically ill patients [18]. In AMI patients, elevated SHR is strongly associated with both 1-year and long-term all-cause mortality [21]. The study by Li et al. [22] demonstrated that the SHR was U-shapedly associated with short-term prognosis and linearly positively associated with long-term prognosis in patients undergoing coronary artery bypass grafting. In patients with diabetes, both higher and lower SHR are associated with adverse renal outcomes [23]. In patients with nonobstructive coronary arteries, higher SHR is associated with an increased risk of rehospitalization for chest pain [24]. Moreover, a meta-analysis of 26 studies (involving 87974 patients) revealed that a higher SHR was significantly associated with an increased incidence of major adverse cardiovascular and cerebrovascular events (HR: 1.7, 95% CI: 1.42–2.03, *P* < 0.001, I^2^: 71%, *P* < 0.01) and in-hospital all-cause mortality (OR: 3.87, 95% CI: 2.98–5.03, *P *< 0.001, I^2^: 54%, *P* = 0.03) [25]. These findings indicate that the SHR may serve as an effective predictor of long-term prognosis. However, these studies primarily focused on the relationship between SHR and adverse outcomes in specific patient populations. In contrast, our study is the first to extend the investigation to a general population of middle-aged and older Chinese adults, demonstrating an L-shaped association between SHR and CVD. This pattern partially aligns with prior research trends and provides new insights into the role of SHR in cardiovascular risk stratification.

Research has demonstrated that elevated SHR is significantly associated with an increased incidence of major adverse cardiovascular and cerebrovascular events, a higher risk of heart failure deterioration, and elevated all-cause mortality [9,26]. However, the L-shaped relationship between the SHR and CVD incidence was observed in our study, where HRs increase significantly when SHR < 0.985, highlighting the impact of low SHR on CVD risk. The SHR is primarily influenced by FBG and HbA1c, with a lower SHR potentially indicating hypoglycemic events or poor chronic glycemic control. Research suggests that recurrent hypoglycemic events are significant risk factors for cardiovascular events [27]. The potential mechanisms by which hypoglycemia leads to cardiovascular damage include the following: (1) hypoglycemia triggers sympathetic-adrenal system activation, causing hemodynamic changes that lead to increased heart rate, enhanced myocardial contractility, and elevated cardiac output, further burdening the heart; (2) hypoglycemia induces abnormal ventricular repolarization, prolonging the QT interval and potentially leading to severe malignant arrhythmias; (3) hypoglycemia activates inflammatory responses, exacerbating endothelial damage, increasing platelet aggregation, and promoting thrombosis, which leads to a higher incidence of embolic events; and (4) hypoglycemia affects myocardial energy metabolism, directly damaging myocardial cells [28–31]. Poor chronic glycemic control over time also significantly increases the incidence of cardiovascular complications. Chronic hyperglycemia leads to endothelial cell damage, accelerates the process of atherosclerosis [32], and increases cholesterol levels in the blood, further exacerbating atherosclerosis risk [33]. Furthermore, hyperglycemia affects the coagulation and fibrinolytic systems, increasing the risk of thrombosis [34]. Therefore, considering previous research, our study emphasizes the clinical importance of maintaining an optimal SHR, as both extremely high and low levels can lead to adverse health outcomes.

The lack of a significant association between SHR and mortality in this study may be attributed to several factors. Firstly, the relatively short follow-up duration may have limited our ability to capture the long-term effects of SHR on mortality. Chronic conditions, including CVD, diabetes, and other metabolic disorders, often have delayed or cumulative effects on mortality, which may not fully manifest within the duration of our follow-up period. The absence of a significant association observed may be influenced by the nature of the CHARLS dataset, which represents a general population rather than a cohort with specific chronic conditions. The heterogeneity of the cohort could obscure the association between SHR and all-cause mortality. Another potential limitation is the presence of residual confounding, as our study may not have accounted for all relevant variables influencing the SHR-mortality relationship. Unmeasured factors, including socio-economic status, lifestyle behaviors (e.g., physical activity, diet, smoking), and long-term medication use, may have contributed to unaccounted variability, thus impacting the observed results. Moreover, SHR may hold prognostic value for subclinical or nonfatal complications not captured in our analysis. Findings by Mone et al. [35] indicate that elevated SHR levels are associated with cognitive decline in frail older adults with heart failure with preserved ejection fraction, potentially mediated by natural killer T-cell activation and microvascular dysfunction. These observations underscore the potential role of SHR as an early marker of organ vulnerability—particularly along the heart–brain axis—rather than a direct predictor of mortality, especially in metabolically vulnerable or aging populations.

The subgroup analysis in this study identified a significant association between SHR and heart disease, whereas no significant relationship was observed with stroke. This contrast may be attributed to the heightened sensitivity of heart disease to metabolic disturbances. These metabolic factors play a critical role in the pathophysiology of coronary artery disease, where processes like endothelial dysfunction, atherosclerosis, and myocardial injury are often exacerbated by abnormalities in glucose metabolism [36,37]. In contrast, stroke is influenced by a broader spectrum of risk factors, including hypertension, small vessel disease, and coagulation abnormalities [38]. Furthermore, the relatively small number of stroke cases in our study cohort may have limited the statistical power to detect a meaningful association between SHR and stroke. The heterogeneity in baseline health conditions within the cohort, encompassing a wide range of comorbidities and health statuses, may have further diluted the association between SHR and stroke.

The similar predictive value of SHR and traditional metabolic markers for all-cause mortality may be attributed to several factors. First, SHR’s primary advantage lies in its ability to capture acute glucose fluctuations triggered by stress, but its clinical utility may be limited in populations not experiencing significant acute stress or critical illness. SHR may not provide additional information in non-acute settings where chronic glycemic control is the predominant risk factor. Therefore, while SHR might enhance cardiovascular risk stratification in patients with SIH, its value may be less pronounced in more stable, long-term patient populations. Second, the basic risk model in our study already incorporated several established cardiovascular risk factors, which accounted for a substantial portion of the variation in mortality risk. Moreover, SHR’s utility may depend on the context in which it is used—while it adds value in acute clinical settings, it may not significantly improve risk prediction in a general population cohort. Finally, the heterogeneity of the CHARLS dataset, which includes a broad range of individuals with varied health statuses and comorbidities, may have diluted the association between SHR and mortality.

## 5 Limitations

This study has several strengths, including the use of a nationally representative dataset, adjustment for multiple covariates, and a comprehensive analysis combining cross-sectional and longitudinal approaches. However, several important limitations should be acknowledged, especially in terms of potential bias and imprecision: First, although the CHARLS dataset provides representative data on middle-aged and older Chinese adults, it may not fully reflect the diversity of the broader population. Regional, socioeconomic, and ethnic heterogeneity—especially among rural or under-resourced communities—could lead to limited external validity. This bias likely results in an underestimation of CVD burden and SHR effects in disadvantaged populations. Second, residual confounding remains possible given the observational design. Despite adjusting for known covariates, unmeasured variables such as dietary intake, stress, medication adherence, and inflammatory biomarkers were not included. The omission of these factors may lead to an overestimation or underestimation of the true association between SHR and outcomes. Future studies using methods such as inverse probability weighting, instrumental variable analysis, or Mendelian randomization could reduce bias from unmeasured confounders. Third, the use of self-reported physician diagnoses for CVD is prone to recall or reporting bias. Misclassification is possible, especially in individuals with limited health literacy. This could dilute the strength of observed associations (bias toward the null) and limit the accuracy of subgroup analyses. Fourth, the mean follow-up duration of 42.2 months may not be sufficient to detect long-term mortality risk. As SHR likely reflects acute metabolic dysregulation, its predictive ability for chronic outcomes such as mortality may require longer observation windows. This limitation may lead to an underestimation of the true hazard over extended periods. Fifth, participants with missing data were excluded, which introduces potential selection bias. If those excluded differ systematically—e.g., poorer health or more advanced disease—this could result in biased effect estimates, likely underestimating the association between SHR and adverse outcomes. Lastly, sex hormones and related metabolic differences were not accounted for, which may be especially relevant given that SHR can be influenced by hormonal status. The failure to stratify or adjust for these factors may mask sex-specific associations and limit generalizability.

## 6 Conclusion

In this study, the index SHR was found to be a valuable index for predicting the risk of CVD in middle-aged and elderly Chinese individuals. There was an L-shaped association between the SHR and CVD risk, in which the inflection point of the SHR for poor prognosis was 0.985. No significant association was observed between the SHR and all-cause mortality, which may be related to sample characteristics or insufficient follow-up time. Despite this, the SHR shows potential as a cost-effective, readily available tool for early CVD risk stratification, particularly in resource-limited settings where advanced biomarkers or imaging techniques may not be accessible. Incorporating SHR measurement into routine clinical practice could enhance early identification of individuals at high risk for cardiovascular events, enabling timely interventions. Further large-scale, multicenter prospective studies should be performed to assess the predictive value of the SHR.

## Supporting information

S1 Table Baseline characteristics of 9008 participants in the longitudinal analysis. (DOCX)

S2 Table Cross-sectional association of SHR with CVD in subpopulations of 12203 participants. (DOCX)

S3 Table Diagnostic performance metrics for identifying all-cause mortality via metabolic markers. (DOCX)

S4 File Raw data. (CSV)

## Abbreviations

AMI: acute myocardial infarction
AUC: area under the curve
BMI: body mass index;
CHARLS: China Health and Retirement Longitudinal Study
CI: confidence interval
CRP: C-reactive protein
CVD: cardiovascular disease
DBP: diastolic blood pressure
eGFR: estimated glomerular filtration rate
FBG: fasting blood glucose
HbA1c: glycosylated hemoglobin
HR: hazard ratio
OR: odds ratio
RCS: restricted cubic spline
ROC: receiver operating characteristic
SBP: systolic blood pressure
SHR: stress hyperglycemia ratio
SIH: stress-induced hyperglycemia
TC: total cholesterol
TG: triglyceride
UA: uric acid
