# Supplementary material for: L-shaped relationship between stress hyperglycemia ratio and cardiovascular disease risk in middle-aged and older adults: Insight from the China Health and Retirement Longitudinal Study
Source: PLoS One. 2025 May 20;20(5):e0324978. doi: 10.1371/journal.pone.0324978 (PMC12091763; doi:10.1371/journal.pone.0324978)
Supplement: S1 Table — (DOCX) [file pone.0324978.s001.docx]

**S1 Table. Baseline characteristics of 9008 participants in the longitudinal analysis.**

| **Characteristics** | **Q1 (≤ 0.739)** | **Q2 (0.739-0.806]** | **Q3 (0.806-0.888]** | **Q4 (> 0.888)** | **Total** | ***P* value** |
| --- | --- | --- | --- | --- | --- | --- |
|  | **n = 2270** | **n = 2242** | **n = 2259** | **n = 2237** | **N = 9008** |  |
| **Demographics** |  | | | | | |
| Age (y) | 60.1 ± 9.4 | 60.1 ± 9.3 | 59.3 ± 9.6 | 59.2 ± 9.5 | 59.7 ± 9.5 | < 0.001 |
| Male (n, %) | 1062 (46.8) | 1019 (45.5) | 1036 (45.9) | 1195 (53.4) | 4312 (47.9) | < 0.001 |
| Marry (n, %) | 2008 (88.5) | 1972 (88.0) | 1993 (88.2) | 1980 (88.5) | 7953 (88.3) | 0.937 |
| **Basic information** |  | | | | | |
| Drinking |  |  |  |  |  | < 0.001 |
| Heavy | 1493 (65.8) | 1475 (65.8) | 1390 (61.5) | 1300 (58.1) | 5658 (62.8) |  |
| Mild | 574 (25.3) | 566 (25.2) | 653 (28.9) | 744 (33.3) | 2537 (28.2) |  |
| Never | 203 (8.9) | 201 (9.0) | 216 (9.6) | 193 (8.6) | 813 (9.0) |  |
| SBP (mmHg) | 126.1 ± 19.9 | 127.4 ± 19.3 | 127.2 ± 19.0 | 128.7 ± 19.1 | 127.3 ± 19.3 | < 0.001 |
| DBP (mmHg) | 74.8 ± 11.5 | 75.4 ± 11.5 | 75.6 ± 11.3 | 76.2 ± 11.6 | 75.5 ± 11.5 | < 0.001 |
| BMI (kg/m^2^) | 23.5 ± 3.6 | 23.7 ± 3.4 | 23.9 ± 3.4 | 24.2 ± 3.6 | 23.8 ± 3.5 | < 0.001 |
| **Comorbidities** |  | | | | | |
| Diabetes (n, %) | 108 (4.8) | 69 (3.1) | 74 (3.3) | 128 (5.7) | 379 (4.2) | < 0.001 |
| Hypertension (n, %) | 354 (15.6) | 394 (17.6) | 365 (16.2) | 407 (18.2) | 1520 (16.9) | 0.071 |
| Dyslipidemia (n, %) | 143 (6.3) | 172 (7.7) | 144 (6.4) | 161 (7.2) | 620 (6.9) | 0.200 |
| Kidney disease (n, %) | 119 (5.2) | 107 (4.8) | 130 (5.8) | 87 (3.9) | 443 (4.9) | 0.029 |
| **Medical treatment** |  | | | | | |
| Diabetes medications (n, %) | 121 (5.3) | 63 (2.8) | 78 (3.5) | 169 (7.6) | 431 (4.8) | < 0.001 |
| Hypertension medications (n, %) | 560 (24.7) | 596 (26.6) | 613 (27.1) | 643 (28.7) | 2412 (26.8) | 0.021 |
| Lipid-lowering therapy | 127 (5.6) | 118 (5.3) | 116 (5.1) | 140 (6.3) | 501 (5.6) | 0.357 |
| **Laboratory data** |  | | | | | |
| TC (mg/dL) | 183.9 ± 37.0 | 185.5 ± 35.2 | 185.1 ± 34.9 | 180.6 ± 36.7 | 183.8 ± 36.0 | < 0.001 |
| TG (mg/dL) | 124.9 ± 77.3 | 131.5 ± 78.8 | 142.4 ± 91.3 | 165.9 ± 106.3 | 141.1 ± 90.5 | < 0.001 |
| eGFR (mL/min/1.73m^2^) | 90.7 ± 15.5 | 91.4 ± 14.9 | 91.3 ± 15.8 | 91.1 ± 17.1 | 91.1 ± 15.8 | 0.107 |
| UA (umol/L) | 4.8 ± 1.4 | 4.9 ± 1.4 | 4.9 ± 1.4 | 5.1 ± 1.4 | 4.9 ± 1.4 | < 0.001 |
| CRP (mg/L) | 2.7 ± 6.6 | 2.3 ± 5.1 | 2.4 ± 5.2 | 2.7 ± 4.9 | 2.5 ± 5.5 | < 0.001 |

The values are presented as the mean ± standard deviation or n (%). A *P* value < 0.05 indicated a significant difference.

Abbreviation: BMI, body mass index; CRP, C-reactive protein; CVD, cardiovascular disease; DBP, diastolic blood pressure; eGFR, estimated glomerular filtration rate; FBG, fasting blood glucose; SBP, systolic blood pressure; SHR, stress hyperglycemia ratio; TC, total cholesterol; TG, triglycerides; UA, uric acid.
