# Supplementary material for: L-shaped relationship between stress hyperglycemia ratio and cardiovascular disease risk in middle-aged and older adults: Insight from the China Health and Retirement Longitudinal Study
Source: PLoS One. 2025 May 20;20(5):e0324978. doi: 10.1371/journal.pone.0324978 (PMC12091763; doi:10.1371/journal.pone.0324978)
Supplement: S2 Table — (DOCX) [file pone.0324978.s002.docx]

**S2 Table. Cross-sectional association of SHR with CVD in subpopulations of 12203 participants.**

| **Outcome** | **OR (95% CI) *P* value** | | |
| --- | --- | --- | --- |
|  | **CVD** | **Heart disease** | **Stroke** |
| SHR | 0.45 (0.32−0.64) < 0.001 | 0.44 (0.30−0.63) < 0.001 | 1.16 (0.60−2.25) 0.655 |
| Age | 1.04 (1.03−1.05) < 0.001 | 1.04 (1.03−1.05) < 0.001 | 1.03 (1.01−1.05) 0.001 |
| Sex | 1.15 (1.00−1.32) 0.055 | 1.24 (1.07−1.43) 0.004 | 0.70 (0.51−0.95) 0.022 |
| Marry | 1.06 (0.90−1.26) 0.472 | 1.05 (0.88−1.25) 0.612 | 1.31 (0.88−1.93) 0.182 |
| Drink | 0.90 (0.78−1.03) 0.137 | 0.98 (0.85−1.14) 0.820 | 0.55 (0.40−0.77) < 0.001 |
| SBP | 1.00 (0.99−1.00) 0.041 | 0.99 (0.99−1.00) 0.005 | 1.00 (1.00−1.01) 0.345 |
| DBP | 1.00 (0.99−1.01) 0.978 | 1.00 (0.99−1.01) 0.750 | 1.00 (0.99−1.02) 0.985 |
| BMI | 1.04 (1.02−1.05) < 0.001 | 1.04 (1.03−1.06) < 0.001 | 0.99 (0.96−1.03) 0.721 |
| Diabetes | 1.15 (0.90−1.48) 0.270 | 1.19 (0.92−1.54) 0.187 | 1.03 (0.62−1.69) 0.923 |
| Hypertension | 3.20 (2.68−3.81) < 0.001 | 3.05 (2.54−3.67) < 0.001 | 2.89 (1.91−4.37) < 0.001 |
| Dyslipidemia | 2.37 (2.03−2.77) < 0.001 | 2.16 (1.84−2.53) < 0.001 | 2.97 (2.18−4.04) < 0.001 |
| Kidney disease | 2.78 (2.32−3.33) < 0.001 | 2.71 (2.25−3.26) < 0.001 | 1.75 (1.19−2.57) 0.004 |
| Diabetes medications | 1.20 (0.94−1.53) 0.142 | 1.14 (0.88−1.46) 0.316 | 1.14 (0.70−1.88) 0.592 |
| Hypertension medications | 1.07 (0.90−1.28) 0.442 | 1.09 (0.90−1.31) 0.375 | 1.12 (0.73−1.70) 0.612 |
| Lipid-lowering therapy | 0.94 (0.76−1.16) 0.568 | 0.97 (0.78−1.21) 0.800 | 0.80 (0.47−1.34) 0.396 |
| TC | 1.00 (1.00−1.00) 0.255 | 1.00 (1.00−1.00) 0.473 | 1.00 (0.99−1.00) 0.112 |
| TG | 1.00 (1.00−1.00) 0.028 | 1.00 (1.00−1.00) 0.006 | 1.00 (1.00−1.00) 0.670 |
| eGFR | 1.00 (0.99−1.00) 0.449 | 1.00 (1.00−1.00) 0.940 | 0.99 (0.98−1.00) 0.019 |
| UA | 0.93 (0.89−0.98) 0.006 | 0.92 (0.88−0.97) 0.002 | 1.02 (0.92−1.13) 0.655 |
| CRP | 1.00 (0.99−1.01) 0.816 | 1.00 (0.99−1.01) 0.762 | 1.00 (0.98−1.02) 0.775 |

A *P* value < 0.05 indicated a significant difference.

Abbreviation: BMI, body mass index; CI, confidence interval; CRP, C-reactive protein; CVD, cardiovascular disease; DBP, diastolic blood pressure; eGFR, estimated glomerular filtration rate; OR, odds ratio; SBP, systolic blood pressure; SHR, stress hyperglycemia ratio; TC, total cholesterol; TG, triglycerides; UA, uric acid.
