# Supplementary material for: L-shaped relationship between stress hyperglycemia ratio and cardiovascular disease risk in middle-aged and older adults: Insight from the China Health and Retirement Longitudinal Study
Source: PLoS One. 2025 May 20;20(5):e0324978. doi: 10.1371/journal.pone.0324978 (PMC12091763; doi:10.1371/journal.pone.0324978)
Supplement: S3 Table — (DOCX) [file pone.0324978.s003.docx]

**S3 Table.** **Diagnostic performance metrics for identifying all-cause mortality via metabolic markers.**

| **Index** | **AUC (95% CI)** | ***P* value** | **Cutoff** | **Sensitivity (95% CI)** | **Specificity (95% CI)** | **Youden’s index** | **PPV (95% CI)** | **NPV (95% CI)** |
| --- | --- | --- | --- | --- | --- | --- | --- | --- |
| FBG | 0.526 (0.485–0.567) | 1(Ref) | 6.259 | 0.832 (0.824–0.839) | 0.253 (0.197–0.310) | 0.085 | 0.977 (0.974–0.981) | 0.038 (0.028–0.047) |
| HbA1c | 0.535 (0.494–0.575) | 0.729 | 6.05 | 0.728 (0.718–0.737) | 0.367 (0.304–0.429) | 0.095 | 0.978 (0.974–0.981) | 0.034 (0.027–0.041) |
| SHR | 0.513 (0.471–0.555) | 0.741 | 0.772 | 0.368 (0.358–0.378) | 0.563 (0.499–0.628) | –0.069 | 0.970 (0.964–0.976) | 0.023 (0.019–0.027) |

Adjusted for age, sex, marital status, BMI, alcohol consumption, mean SBP and DBP, hypertension, dyslipidemia, diabetes, chronic kidney disease, use diabetes medications, use hypertension medications, lipid-lowering therapy, TC, TG, eGFR, UA, and CRP. A *P* value < 0.05 was considered statistically significant.

Abbreviations: AUC, area under the curve; BMI, body mass index; CRP, C-reactive protein; DBP, diastolic blood pressure; eGFR, estimated glomerular filtration rate; NPV, negative predictive value; PPV, positive predictive value; ROC, receiver operating characteristic; SBP, systolic blood pressure; SHR, stress hyperglycemia ratio; TC, total cholesterol; TG, triglyceride; UA, uric acid.
